# Supplementary material for: Complexome profiling of the Chlamydomonas psb28 mutant reveals TEF5 as an early PSII assembly factor
Source: Plant Cell. 2025 Mar 17;37(6):koaf055. doi: 10.1093/plcell/koaf055 (PMC12164590; doi:10.1093/plcell/koaf055)
Supplement: koaf055_Supplementary_Data [file koaf055_supplementary_data.zip › psb28_interactive.zip!/plots/intensity/qualityPlots/peptides/PLAP4_Cre12.g502250.t1.2.html]

### Cre12.g502250.t1.2

Trivial name: PLAP4  
SeqCoverage [median]: 13.10  
Mass: 39.79 kDa  
  
 

| Sequence | MissedCleavage | Mass [Da] | wtIntensity | muIntensity | Score |
| --- | --- | --- | --- | --- | --- |
| GGAADAAGMDQVGLQR | 0 | 1515.71 | 385.34 | 415.22 | 140.14 |
| KEEAGGGAEAGAGGVTPAAA | 1 | 1669.79 | 282.84 | 487.63 | 58.32 |
| TSSVVLSTTYLDER | 0 | 1569.79 | 0.00 | 990.04 | 100.72 |
|  |  |  | 668.2 | 1892.9 |  |
